# Therapeutic education programme in TTR-FAP

**DOI:** 10.1186/1750-1172-10-S1-I24

**Published:** 2015-11-02

**Authors:** Marie Théaudin, Cécile Cauquil, Teresa Antonini, Vincent Algalarrondo, Céline Labeyrie, Sophie Aycaguer, Marie Kubezyk, Colombe Lemoine, Géraldine Nonnez, Agnès Morier, Nadia Kouchi, Amandine Darras, Nathalie Coste, Mireille Clément, David Adams

**Affiliations:** 1 French Reference Centre for Familial Amyloid Polyneuropathy, NNerf, Neurology Department, Hôpital Bicêtre, Assistance Publique Hôpitaux de Paris (APHP), HUPS, Le Kremlin-Bicêtre, France; 2 Hôpital Bicêtre, APHP, Neurology Department, APHP, HUPS, Villejuif, France; 3 INSERM UMR788, Université Paris Sud, Paris, France; 4 Hôpital Paul Brousse, Centre Hepato-Biliaire, APHP, HUPS, Villejuif, France; 5 Hôpital Antoine Béclère, Cardiology Department, APHP, HUPS, Clamart, France; 6 Université Paris Sud, Paris, France; 7 EDU-santé, Vanves Cedex, France; 8 Association Française contre l’Amylose, Aix en Provence Cedex 3, France

## Background

The French National Reference centre for Transthyretin-related amyloidosis (ATTR) was accredited in 2005. One of its 10 lines of action is to inform and educate patients about their disease to improve their care and reduce morbidities. We thus decided to elaborate a therapeutic patient education (TPE) programme, starting with patients’ needs assessment. We will describe our needs assessment and discuss our experience in TPE for ATTR patients.

## Methods

A qualitative research study was conducted with one-to-one semi-structured interviews of selected individuals. Recorded interviews were analysed to identify the skills that patients need to acquire. A TPE programme was elaborated on the basis of these findings.

## Results

Analysis of the interviews showed that interviewees had a good knowledge of the disease and its symptoms but they had difficulties explaining the disease mechanism and did not have an adequate knowledge of the available treatment options, although they knew that liver transplant might halt progression of the disease. ATTR amyloidosis appeared to have a major negative impact on the patient’s physical and mental well-being. Patients feared loss of autonomy and having to require assistance from their relatives and spouses. All interviewees were keen to participate in a TPE programme. Based on this needs assessment, we identified seven skills that patients need to acquire and several pedagogical goals to be achieved during the education programme. An interdisciplinary team then elaborated a complete TPE programme and its educative tools. The programme includes 2 individual sessions (initial educational diagnosis assessment and final assessment of the skills acquired during the programme) and 7 collective sessions. First TPE sessions started in October 2014. To date, 8 patients living close to Paris attended 4 collective TPE sessions. After the sessions, all patients stated they were very satisfied with the quality of the education provided, the educational tools, the timing of the sessions. They all stated the collective sessions met their expectations.

## Conclusion

Elaboration of a TPE programme for ATTR amyloidosis required to obtain useful information from the patients themselves, and their relatives, concerning their perception of their disease. This needs’ assessment constituted the basis for designing the first TPE programme, to our knowledge, for ATTR amyloidosis. Patients who attended the first TPE sessions were very satisfied and felt that the sessions fully met their educational expectations. Further TPE sessions are scheduled within the end of 2015 and we plan to organize TPE sessions in other French cities where ATTR amyloidosis patients are followed.

